# Public Pharmacists' "Open Evening"

**Published:** 1915-04-10

**Authors:** 


### Public Pharmacists' "Open Evening."

DR. R. L. SHERLOCK, D.Sc., ON "GEOLOGY AND SCENERY."

A meeting of the Public Pharmacists' and Dispensers' Association was held on Wednesday, March 31, 1915, and took the form of an "open evening" for members and their friends.

The lecture theatre of the Pharmaceutical Society of Great Britain at Bloomsbury Square, London, was kindly placed at the disposal of the Association by the President and Council of that body. Mr. J. Hassall France presided over an exceptionally good gathering, which included Mr. and Miss Lindsey, Mr. and Mrs. Welford, Mr. and Mrs. Jenkin, Mr. W. Kinsman, Mrs. France, Mrs. E. France, Mr. and Mrs. Heworth, Mr. C. A. Allgood, Mr. E. E. Smith, Mr. and Mrs. Wells, Misses C. Andrews, Gilliatt, Hughes, Sproule, Kaye, and Messrs. T. Goodall, Elias, Lindsay, and others.

Dr. R. L. Sherlock, D.Sc., F.G.S., gave a lecture on "Geology and Scenery." The formation and structure of rocks was explained, and the different stages in the "heading" and breaking-off of rocky structures was demonstrated by a series of fine lantern slides. The processes in the formation of "gorges," etc., by "faults" in and contraction of rocks was also shown. Dr. Sherlock then gave an interesting account of lava formation. The effect of rain and atmosphere was also dealt with. Methods of the production of volcanoes—due to reduced pressure of top layers of earth, thus allowing the molten interior contents to escape as a superheated jet, which dislodges rock, etc., at the earth's outer surface—were graphically demonstrated.

The lecturer then explained the part which glaciers played in the shaping of rocky matter, illustrating this with some particularly good lantern views. Altogether some fifty or sixty slides were shown, which, besides being descriptive, were photographically all that could be desired.

Mr. Gibson, in proposing a vote of thanks to Dr. Sherlock, referred to the inter-dependence of the sciences on each other, and said that chemists and pharmacists could elucidate many problems by having some knowledge of the other sciences, such as botany, zoology, and geology. Mr. R. Welford seconded the vote, which was cordially endorsed by those present. The many institutional pharmacists and their friends present were obviously delighted with this evening meeting arranged by the Association.

